# Mineral Content of Liver of Buffaloes (*Bubalus bubalis*) Reared in Different Ecosystems in the Eastern Amazon

**DOI:** 10.3390/ani13071157

**Published:** 2023-03-25

**Authors:** Laurena Silva Rodrigues, Jamile Andréa Rodrigues da Silva, José de Brito Lourenço-Júnior, André Guimarães Maciel e Silva, André Martinho de Almeida, Miguel Pedro Mourato, Vinicius Costa Gomes de Castro, Welligton Conceição da Silva, José António Mestre Prates

**Affiliations:** 1 Postgraduate Program in Animal Science (PPGCAN), Institute of Veterinary Medicine, Federal University of Para (UFPA), UFRA, Brazilian Agricultural Research Corporation (EMBRAPA), Castanhal 68746-360, Brazil; 2 Institute of Animal Health and Production, Federal Rural University of the Amazônia (UFRA), Belem 66077-830, Brazil; 3 Linking Landscape, Environment, Agriculture and Food (LEAF), Institute of Agronomy (ISA), University of Lisbon, 1349-017 Lisboa, Portugal; 4 Postgraduate Program in Animal Health and Production in the Amazon (PPGSPAA), Federal Rural University of the Amazon (UFRA), Belem 66077-830, Brazil; 5 Center for Interdisciplinary Research in Animal Health (CIISA), Faculty of Veterinary Medicine, University of Lisbon, 1300-477 Lisboa, Portugal; 6 Associate Laboratory for Animal and Veterinary Science (AL4Animals), 1300-477 Lisboa, Portugal

**Keywords:** macro and micro minerals, ruminants, Amazonia, water buffalo, native pasture

## Abstract

**Simple Summary:**

In recent years, the search for a healthy standard of living has become imperative, so consumers seek more information about the nutritional composition of the foods they consume. In this context, there is a deficiency in the characterization of the mineral components present in the liver of buffaloes. Thus, the aim of the study was to evaluate the mineral content of liver from buffaloes raised in different production ecosystems; three in native and cultivated pastures, in two times seasons of the year (dry and rainy season), and one in confinement in the Eastern Amazon. The different ecosystems of native and cultivated pastures, in the dry and rainy periods of the year, and one in an intensive ecosystem in the Eastern Amazon, influenced the levels of minerals present in the liver tissue of buffaloes. The different ecosystems studied affected the values of minerals found in the liver of buffaloes raised in these ecosystems, for sodium (Na), K, calcium (Ca), magnesium (Mg), sulfur (S), copper (Cu), iron (Fe), manganese (Mn) and barium (Ba). Therefore, buffalo liver is an excellent source of minerals and can be included in the human diet, in order to meet nutritional requirements for minerals.

**Abstract:**

This study aimed to evaluate the influence of different production ecosystems, three in native and cultivated pastures (extensive), at two seasons of the year (dry and rainy), and one in confinement (intensive) in the Eastern Amazon, on the mineral content of buffalo liver raised on these ecosystems. Twelve male buffalo (*n* = 12), aged between 24 and 36 months, slaughtered in commercial slaughterhouses, were used in each of the ecosystems considered: Marajó; Lower Amazon; Cultivated Pasture, and in confinement system, Pará, Brazil. Approximately 5 g of liver was collected, stored and frozen until lyophilization. Samples were analyzed for mineral content based on inductively coupled plasma optical emission spectrometry (ICPOES) readings. The relationship between extensive ecosystems and an intensive production system (*p* < 0.05) in the values of potassium (K), iron (Fe), copper (Cu), manganese (Mn) and barium (Ba) was evaluated. The different ecosystems studied influenced (*p* < 0.05) the mineral values found in the liver of buffaloes raised in the ecosystems, for sodium (Na), K, calcium (Ca), magnesium (Mg), sulfur (S), copper (Cu), iron (Fe), manganese (Mn) and barium (Ba). The period of the year interacted with the values of Na, K, S and Cu; however, an interaction of local vs. period of the yer was observed for the values of K, Mg, P, S and Cu. It can be concluded that the buffalo liver is an excellent source of minerals and can be included in the human diet and that the ecosystem the animals are raised influences its content.

## 1. Introduction

The water buffalo species has shown to be increasingly efficient in terms of productivity, in addition to improving its ability to adapt to environments, while the appreciation of the nutritional value of its meat and other marketed organs has pleased the population that consumes them [1,2,3,4,5,6,7,8,9].

Among the various nutrients contained in the liver of ruminants, minerals are of fundamental importance, as they participate in multiple vital functions of the animal and human organism [10,11,12,13,14,15]. The animal organism contains around 50 minerals; macrominerals, which are calcium, phosphorus, magnesium, potassium, sodium, chlorine and sulfur, which are required in greater quantities, and microminerals such as iron, cobalt, copper, iodine, manganese, zinc and selenium, which are called essential, as they are needed in small amounts [16,17].

Dietary Reference Intakes (DRIs) developed by the Food and Nutrition Board (FNB) at the Institute of Medicine [18] of the National Academies (formerly the National Academy of Sciences) reported that beef liver provides around 7.5 mg of Fe/85 g, being the second largest source of iron among meat products, with chicken liver in the first position, per portion. The recommended Fe content per individual was considered according to age, diet, sex, among others. A premenopausal woman, for example, needs 18 mg of Fe per day.

In the Eastern Amazon, buffaloes are raised in pasture ecosystems, such as native pastures from Marajó, native pastures from Lower and Middle Amazon, and from native and cultivated pastures from drylands, where more productive foragens with better nutritional value are used, in herds formed by animals with a better genetic pattern [19,20,21]. Buffaloes use these pastures more efficiently than cattle [22]. It is believed that the different Amazonian ecosystems can influence the levels of minerals in the liver of these animals. The aim of the study was to evaluate the mineral content of liver from buffaloes raised in different production ecosystems; three in native and cultivated pastures, in seasons (dry and rainy), and one in confinement in the Eastern Amazon.

## 2. Materials and Methods

### 2.1. Animals

The study was carried out using samples of liver from crossbred buffaloes of the Murrah/Mediterranean breeds, from three breeding ecosystems in the Eastern Amazon, raised in extensive production systems, in two seasons of the year (dry and rainy season), and animals raised in confinement, in an intensive production system.

After the animals had been slaughtered in a commercial slaughterhouse using commercial practices, a sample of the liver and adipose tissue of twelve animals reared in different ecosystems was obtained. Each farm was considered a representative sample of the production system in that particular region. Thus, this research was approved by the Ethics Committee for the Use of Animals (CEUA), with protocol number 4542190820, granted by the Federal Rural University of Amazônia (UFRA).

These tissue samples were collected in commercial slaughterhouses, where the animals were slaughtered, in each of the considered ecosystems. 

For each treatment were used twelve male buffaloes, aged between 24 and 36 months, with an average weight of 432 kg at end rainy and 409 kg at end dry season. The animals of confinement system tinha 18 months and average weight of 433 kg. The total of animals added 84.

### 2.2. Ecosystems Studied

The experimental treatments involved three under pasture buffalo-breeding ecosystems in the Amazon, and one under confinement for slaughter, in an intensive system:

Ecosystem 1—Marajó—rural breeding property located in the Mesoregion of Marajó, Soure, Pará, Brazil (latitude 0°39′27.89″ S, longitude 48°42′35.01″ W, altitude of 7 m). Tropical rainy climate Am, according to the classification of Kööpen [23], with an average annual temperature of 27 °C, average relative humidity of 85% and average annual precipitation of 2500 mm, distributed in two distinct periods: one of maximum, January to June (average temperature—AT of 27.7 °C, maximum temperature—MaxT of 31.7 °C, minimum temperature—MinT of 22.7 °C, RH of 87.2% and average rainfall—AR of 316.4 mm), and another from minimum, from September to November (AT of 28.8 °C, MaxT of 32.9 °C, MinT of 23.3 °C, RU of 83.0% and AR of 122.2 mm) [24]. In this ecosystem, buffaloes are reared in a traditional system, fed exclusively on pasture, with grasses native to areas subject to flooding, such as *Panicum elephantipes, Leersia hexandra and Hymenachne amplexicaulis*. In this system, the animals received supplemental mineral salt *ad libitum*.

Ecosystem 2—Lower Amazon - rural property located in the Lower Amazon Mesoregion, Santarém, Pará, Brazil (latitude 02°41′48.83″ S, longitude 54°38′35.43″ W, altitude 108 m). Rainy tropical Am climate, according to the Kööpen classification, with an average annual temperature of 26 °C, average relative humidity of 86% and rainfall of 2000 mm/year, with greater intensity between January and May (AT of 26.1 °C, MaxT of 29.8 °C, MinT of 23.8 °C, RH of 87.6% and AR of 296 mm) [24], when average precipitation monthly ranges from 120 mm to 380 mm. The months of June to November (AT of 27.2 °C, MaxT of 31.4 °C, MinT of 24.2 °C, RH of 84.4% and AR of 20 mm) [24] are the driest period, with average rainfall below 60 mm. In this breeding ecosystem, buffaloes are raised in the traditional way on pasture, in native pastures in areas subject to flooding. During the collection of pastures, there was also an availability of cultivated grasses Panicum maximum cv. Mombasa and *Bhachiaria brizantha*. In this system, the animals received supplemental mineral salt *ad libitum*.

Ecosystem 3—Cultivated Pasture —rural property located in the Northeast Mesoregion of Pará, Nova Timboteua, Pará, Brazil (latitude 01°12′52.63″ S, longitude 47°24′30.94″ W, altitude 53 m). Am-type climate, according to the Kööpen classification, average annual temperature of 26.1 °C, average relative humidity of 86% and average annual rainfall of 2467 mm, with a dry period from September to November (AT of 29.1 °C, MaxT of 33.5 °C, MinT of 22.0 °C, RU of 75.2% and AR of 25.1 mm) [24] and a rainy period from December to August (AT of 27.2 °C, MaxT of 31.9 °C, MinT of 22.5 °C, RU of 86.1% and AR of 259.7 mm) [24]. In this ecosystem, the animals was feed on cultivated drylands pastures (*Panicum maximum* cv. Mombaça and *Bhachiaria humidicola*), in areas originally forested. In this system, the animals received supplemental mineral salt *ad libitum*. The animals received Wet Brewery Waste during the dry period.

Ecosystem 4—Confinement—rural property located in the Northeast Mesoregion of Pará, Tomé-Açu, Pará, Brazil (latitude 02°25′08″ S, longitude 48°09′08″ W, altitude 45 m). In this breeding system, the animals, called “Buffalo Premium”, are confined with initial weight around 218 kg and left with 433 kg, at 18 months of age, after 192 days of feeding. Feed consisted of sorghum silage, soybean meal, wet sorghum premix and commercial feed (high performance core).

### 2.3. Collection of Food Samples and Bromatological Analysis

Pasture samples were collected from rural properties where the experimental animals were raised. Subsamples of 1 m^2^ were collected at five different points in each pasture. Subsequently, the five subsamples from each pasture were homogenized together, weighed, and then approximately 1 kg of sample from each forage was collected, which were sent separately to the laboratory. Food samples offered to animals in the confinement system were also collected. Bromatological analyses were carried out at the Laboratory of Animal Nutrition at the Federal University of Pará/Campus Castanhal.

The feed samples were subjected to partial drying in a forced ventilation oven at a temperature of 55 to 60 °C for 24 to 72 h, to avoid loss of volatile compounds and chemical changes, allowing the analysis of their components later. After drying, the samples were cooled to room temperature in order to minimize moisture changes and ground in a Willey mill to 1 mm for laboratory analysis. Food samples were analyzed for dry matter (MS; method INCT-CA G-003/1) at 105 °C for 16 h and mineral matter (MM; INCT-CA M-001/1) in a muffle furnace at 600 °C for 4 h.

Total nitrogen (INCT-CA N-001/1 method) was quantified using a three-step micro Kjeldhal analysis (digestion with sulfuric acid, basic distillation and titration with hydrochloric acid) and the value obtained was multiplied by 6.25 to obtain crude protein (CP).

The contents of ether extract (EE; INCT-CA G-004/1), neutral detergent fiber (NDF; INCT-CA F-001/1 and INCT-CA F-002/1) and acid detergent fiber were evaluated, as well as acid (ADF; INCT-AC F-003/1 and INCT-AC F-004/1), both corrected for protein and ash, according to the methods recommended by the National Institute of Science and Technology in Animal Science (INCT-CA) [25].

To determine non-fiber carbohydrates, the methodology of Sniffen et al. [26] and for TDN.

### 2.4. Collection of Animal Tissue Samples

Twelve liver were collected from water buffaloes belonging to each experimental group (ecosystems and period of year). The sampling was carried out upon arrival at the slaughterhouse, after evisceration. Approximately 50 g (wet weight) of tissue were collected per sample and stored in a freezer and frozen at −80 °C. Then, the samples were lyophilized for a minimum of 24 h (liver), using a Christ Alpha 1–2 LDplus lyophilizer (Christ alpha, Osterode am Harz, Germany) in the Laboratory of Animal Nutrition, Federal Rural University of the Amazon, Belém, Pará, Brazil. The lyophilized samples were sent to the Faculty of Veterinary Medicine, University of Lisbon, where they were processed.

### 2.5. Sample Preparation and Digestion

Approximately 1 g of the liver tissue was weighed and dried to constant weight at 65 °C. After drying, all samples were ground with a mortar and pestle until obtaining a fine and homogeneous powder and weighed in a digestion tube (50 mL) until an approximate weight of 0.3 g.

Sample dissolution was performed as an adaptation of the method described by Roselli et al. [27]. Briefly, 3 mL of concentrated nitric acid, 10 mL of acid hydrochloride and 1 mL of hydrogen peroxide were added to each digestion tube. The samples were left in the acids for 16 h and hydrogen peroxide was added immediately before digestion to avoid sample loss due to the reaction between the hydrogen peroxide and the acids.

After adding the acid mixture, the tubes were randomly distributed on a digestion plate (DigiPREP MS, SCP Science) in which they were heated following the pattern: 1 h to reach 95 °C and then 1 h at 95 °C. After a total digestion time of 2 h, the samples were allowed to cool in a ventilated chamber.

Once at room temperature, the samples were diluted with distilled water in a volumetric flask (25 mL). Diluted samples were filtered into sealed vials using filter papers with a diameter of 90 mm (Filter-Lab ref. 1242, Filtros Anoia, S.A., Barcelona, Spain). Since ICP-OES readings could occur, the filtered solution was transferred to the ICP tubes. These tubes were then arranged with their respective blanks on a conveyor.

### 2.6. Readings by Inductively Coupled Plasma Optical Emission Spectrometry (ICP-OES)

ICP-OES readings were performed on a Thermo Scientific iCAP 7000 series, ICP-OES spectrometer, equipped with an autosampler. The standards of various elements were used to create the necessary calibration curves to quantify the different elements. Detection and quantification of various elements took place overnight, to detect the following elements: Sn (tin), V (vanadium), Li (lithium), Ba (barium), Se (selenium), As (arsenic), Co (cobalt), Zn (zinc), Fe (iron), Mn (manganese), Cu (copper), Pb (lead), Cd (cadmium), Ni (nickel), Cr (chromium), S (sulfur), P (phosphorus), Mg (magnesium), Ca (calcium), K (potassium) and Na (sodium). No further dilution was required for any element prior to analysis.

### 2.7. Statistical Analysis

The experimental design was completely randomized in a 3 × 2 + 1 factorial arrangement (three ecosystems x two seasons of the year and one additional treatment corresponding to confinement). The collected variables were analyzed in PROC MIXED of SAS version 9 [28], considering the following model: Yijk = µ + αi + βj + γĳ + ԑĳk and yh = µ + δα + Ԑh

in which:

Yijk: response variable related to the i-th level of the ecosystem (i = 1, 2 and 3) with the j-th level of the period (j = 1, 2) in the k-th repetition (k = 1, 2, …, r);

µ: overall mean;

αi: effect of the i-th level of the ecosystem;

βj: effect of the jth level of the period;

γĳ: interaction effect of the i-th level of the ecosystem with the j-th level of the period;

ԑĳk: experimental error associated with the animal of yijk and assumption that ԑĳk ~ N (0, σ2) is independent;

yh: response variable associated with the h-th observation (h = 1, 2, …, m) of additional treatment;

δα: is the effect of additional treatment;

Ԑh: experimental error associated with additional treatment with the assumption that Ԑh ~N (0, σ2) is independent.

The model considers ecosystems and seasons as fixed effects (meat produced extensively or intensively). Contrasts between extensive and intensive ecosystems and interaction between experimental site and time of year were considered. To assess differences between groups, at a significance level of 0.05, the Tukey–Kramer test was applied, as the data were unbalanced due to losses.

## 3. Results

Table 1 shows the chemical and mineral composition of the diets of Murrah vs. Mediterranean buffaloes raised intensively (confined) and extensively in three types of ecosystems in the Amazon, Brazil, in the dry and rainy periods of the year.

The values of minerals contained in the liver tissue of buffaloes rearing in different ecosystems in the Eastern Amazon, in the dry and rainy period of the year, and one in the confinement ecosystem are in Table 2.

The different ecosystems studied significantly influenced (*p* < 0.05) the results obtained for Na contained in the liver. There was a significant influence (*p* < 0.05) of the location, with higher values of Na, for the dry season of 3111.65 mg/kg and for the rainy season of 3497.28 mg/kg in the liver of buffaloes raised on Marajó. The periods of the year also influenced the results, with higher Na values in the liver tissue of the animals reared during the rainy season of the year; however, there was no difference between the intensive and extensive systems (*p* > 0.05) (Table 2).

The extensive vs. intensive ecosystem relationship showed significant differences (*p* < 0.05) for the K present in the liver of buffaloes. The place of study, the period of the year and the interaction place vs. period of the year influenced the results detected in the liver for K (Table 2).

The results of Ca were influenced (*p* < 0.05) by the different ecosystems, with higher values in the liver tissue of animals reared in all ecosystems, except for the Cultivated Pasture ecosystem (Table 2). The different study sites significantly influenced (*p* < 0.05) the results of Mg contained in the liver of buffaloes raised in these ecosystems. The location vs. season of the year interaction significantly influenced the values of P, present in the liver of buffaloes. The location, the time of year and the location vs. season of year interaction significantly influenced (*p* < 0.05) the values of S present in the liver of buffaloes raised in the different ecosystems.

The extensive vs. intensive ecosystem relationship, location, period of the year and the interaction location vs. season of the year significantly influenced (*p* < 0.05) the Cu results observed in the liver tissue of buffaloes. The results of Zn present in the liver of buffaloes raised in different Amazonian ecosystems were different (*p* < 0.05). The relationship between extensive and intensive ecosystems and the study site influenced the results of Fe contained in the liver. The relationship between extensive and intensive ecosystems and the studied locations influenced the effects obtained for Mn, with higher values observed in the ecosystem of the Marajó. The relationship between extensive and intensive ecosystems and the study site significantly influenced (*p* < 0.05) the Ba values present in buffaloes’ livers (Table 2).

There was no effect of ecosystems and seasons on the values of the Cr, Ni, As, Li, Pb, Co, Sn, V and Cd. Cr and Ni showed values lower than 2.0 mg/kg in dry matter (DM) in the liver tissue of buffaloes from the different Amazonian ecosystems studied. As and Li showed values lower than 1.5 mk/kg. Pb, Co, Sn and V showed values lower than 1.0 mg/kg and Cd showed values lower than 0.05 mg/kg in DM in the liver of buffaloes from the different ecosystems studied. 

## 4. Discussion

Among the minerals, the Na is an important mineral for human nutrition [29]. Along with potassium, it regulates the body’s water balance. It influences the conduction of nerve impulses, muscle contractions and blood pressure [30]. The highest Na values were found in the liver of buffaloes raised in the Marajó ecosystem, at the end of the dry season, 3111.65 mg/kg and at the end of the rainy season, with greater availability of forage mass, 3497.28 mg/kg (Table 2). This may have occurred because higher levels of Na were observed in the pasture on Marajó in both periods studied (DR = 1288.62 and RS = 2956.53). In addition, the buffaloes in this ecosystem fed on three different sources of grass (*Panicum elephantipes*, *Leersia hexandra* and *Hymenachne amplexicaulis*), which may have favored the increase in this mineral in the livers of the studied animals. In addition, Marajó water contains higher levels of Na, as it is brackish. In the analysis of the concentration of this macromineral in the liver of lambs of three different breeds (Australian Merino, Damara and Dorper), values [30] close to those found in the present study were observed.

Associated with Na and K acts in the body’s water balance, acts in the transmission of nerve impulses, maintains normal heart rate and blood pressure [31]. For K, the lowest value was detected in the liver of animals raised in the dry season at lower amazon (10,454.0 mg/kg) and the highest (12,051.0 mg/kg) in the liver of buffaloes raised at cultivated pasture, during the dry season. of the year, Such results can be explained by the influence of the time of year on the absorption of this mineral, as the observed effects occur because this element is an intracellular ion capable of providing considerable muscle stimuli [32]. In Lower Amazon, even with high concentrations of K in the forage in the dry period (K = 18,355.04 mg/kg), the lowest levels of this mineral were observed in the liver, which may be related to the low rate of ingestion of these forages in the dry period of the year. In cultivated pasture, where a higher amount of K was observed in the liver of buffaloes, in addition to the considerable amount of this mineral in the forage (K—18,759.52 mg/kg), that there was a higher consumption rate, which may have favored the higher concentration of this mineral in the liver.

Among the minerals, the Ca is the most abundant mineral in the body. Approximately 99% is present in bones and teeth, the rest remaining is found in other tissues. It acts in balance with phosphorus and is essential for the maintenance of bone tissue. It participates in the regulation of blood pressure, blood clotting, muscle contraction, hormone secretion, nerve transmission and, together with phosphorus, forming the structure of several enzymes [33]. The lowest Ca values were observed in the liver tissue of buffaloes raised in the cultivated pasture ecosystem, in both periods of the year (Table 2). Although the cultivated pasture pasture has higher levels of Ca (DR = 5413.91 mg/kg, RS = 6536.74 mg/kg), there was probably less absorption of this mineral in the intestines of the animals. This Ca absorption may be related to vitamin D deficiency [34], which was not measured in this work. The highest concentration of Ca in the liver of buffaloes raised on Marajó in both periods of the year may be associated with a diet rich in this mineral (DR = 2672.26 mg/kg, RS = 2624.79 mg/kg) with high rates of forage consumption.

The Ca content in the liver of buffaloes from all ecosystems studied was higher than the values found by Tajik et al. [35], who reported Ca content in the liver of male buffaloes from Iran, 219.90 mg/kg, a much lower value than those found in the present study. It seems that the mineral composition of the diet influences the mineral bioavailability and, consequently, the mineral profiles of the tissues studied [14]. It was observed in supplemented castrated kids and lambs from the Polish plain, an increase in Ca concentrations in the liver of these animals, 6.66 mg/kg and 15.24 mg/kg, respectively [36,37], which are relatively low values when compared to the present study (Table 2).

The Mg participates in the formation of bones and teeth, it is essential in muscle contraction and relaxation, it also participates in the immune system, in the formation of antibodies and in the activation of several enzymes [33]. The livers of animals reared in the lower amazon ecosystem, during the rainy season of the year, had the highest Mg value (731.72 mg/kg). The liver tissue of buffaloes raised in the cultivated pasture ecosystem showed higher P values at the end of the dry period (Table 2). The diet consumed by animals in this ecosystem, as shown in Table 1, showed higher levels of P. S was relatively higher in the livers of animals reared in the ecosystems of lower amazon, in the rainy season (6703.85 mg/kg), on the Marajó in both periods (dry period, 6687.72 mg/kg and rainy period, 6798.98 mg/kg) and in confinement (6555.97 mg/kg). Similar results for Mg levels were evidenced by Ribeiro et al. [30] in the liver of lambs of three breds. These mineral differences, evidenced according to the region evaluated, indicate that the nutrition from each forage present in the three different ecosystems influences the mineral content present in the animals [38,39,40,41,42,43,44]. The highest amount of Mg evidenced in lower amazon in the rainy period (RS = 3998.71 mg/kg) and in Marajó in the dry season (DR = 2226.14 mg/kg) may have occurred due to the presence of high concentrations of this mineral in the forage of these ecosystems, 3998.71 mg/kg and 2226.14 mg/kg, respectively.

Like Ca, P is vital for building strong bones and teeth. It builds the structure of cells and is important in many biochemical reactions, such as energy metabolism. The amounts of phosphorus and calcium need to be in balance with each other for them to perform their functions [43]. The daily P recommendation for adult humans is 700 mg [44]. The liver tissue of buffaloes reared in the cultivated pasture ecosystem, in the dry period, showed a higher content (12,140 P mg/kg/DM) for P (Table 2) and lower for lower amazon in the rainiest period (12,010 P mg/kg/DM). This may have occurred due to the high concentration of this mineral in the forage of the studied ecosystem (DR—2426.34 mg/kg, Forage 2772.62 mg/kg) when compared with Marajó.

The animals from Marajó had the lowest P values in the liver (DR = 11,974 mg/kg, RS = 11,469 mg/kg), even with high levels of this mineral in the ingested forage in both periods (DR = 2030.83 mg/kg, RS = 806.84 mg/kg). probably because this is an intracellular anion, acting in energy storage, transport and the structuring of cell membranes, in addition to participating in the structure of bones and teeth, the structure of the cell nucleus and cytoplasm, acid-base balance, transforming energy, transmitting impulses and participating in the metabolism of carbohydrates, fats and proteins [45]. These activities may have required part of the P present in the liver, thus reducing its concentration in this organ. Another justification for the low concentration of P in the liver is the interaction of this mineral with Ca, and the higher Ca content in the diet, as observed in this study, may have caused a reduction in P in the body [46,47]. In Brazil, P deficiency in animals is the most common and economically important disease [48]. 

Among the minerals, Cu plays an important role in the immune system, as it increases the feedback for monocytes, neutrophils and T cells [49], and its deficiency is related to a reduction in the cellular and humoral immune response [50]. Another important point is that it is associated with the maturation of red blood cells, in addition to of a having a synergistic effect with iron in the formation of hemoglobin [51]. This microelement participates in the formation of bone and connective tissue and is important in the integrity of the central nervous system [52]. Among the microminerals studied, Cu was found in greater amounts in the liver of buffaloes raised in the cultivated pasture ecosystem in the dry period of the year (257.96 mg/kg) and the wettest period of the year (93,784.0 mg/kg). This value is much higher than that found by Pereira and Cardoso [53], who observed mean liver copper values of 19.51 mg/kg in young and adult buffaloes raised on the Marajó. Copper levels in the liver tissue of 104 buffaloes were also evaluated on the Marajó, which showed mean values of 5.57 ± 7.60 mg/kg [52]. Buffaloes from cultivated pasture consumed forage with higher Cu content both in the dry (DR = 6.70 mg/kg) and rainy season (RS = 6.09 mg/kg) when compared to the ecosystems of lower amazon (DR = 3.19 mg/kg, RS = 3.08 mg/kg) and Marajó (DR—Not identified, RS = 3.63 mg/kg), maximizing the concentration of this mineral in the livers of the animals. The daily Cu recommendation for human food is 0.9 mg; therefore, buffalo livers from all ecosystems meet the recommendations.

The Zn is essential for DNA synthesis and for the development, enhancement and maintenance of the immune system, in addition to being an important enzymatic cofactor for more than 300 metalloenzymes. It participates in the catalytic activity of enzymes involved in the metabolism of carbohydrates, lipids and proteins [10,45,54]. The highest value of Zn (116.44 mg/kg) was found in the liver of buffaloes from the Marajó ecosystem, this may have occurred due to the high amount of this mineral in the forage of this ecosystem (37.07 mg/kg). Contrary to what was detected in the work in question, mean values of Zn (27.05 mg/kg ± 13.12) were found in buffalo liver tissue, also on the Marajó, with lower values and were thus considered deficient [45]. Daily recommendations for zinc for human consumption is 7 mg.

The Fe is one of the components of hemoglobin in red blood cells and is essential for transporting oxygen to the body. Although their requirements are small, it is very important to consume food sources, remembering particularly as that women need, on average, twice as much iron in the diet as men [55]. The highest Fe value (1381.29 mg/kg) was found in the liver of buffaloes from the Marajó ecosystem in the RS period (Table 2), this can be explained by the high Fe content in the food consumed by the animals in the RS period (1750.79 mg/kg) in this ecosystem, as shown in Table 1, maximizing the amount of this mineral in the buffalo liver. In addition, the more intense the activity developed by the animal, the greater the need for proteins present in Fe, such as myoglobin [56,57]. The daily recommendation for Fe intake for humans is 14 mg.

Among the minerals, the Mn is important for the production of some enzymes, participates in the formation of bones and tendons and has an antioxidant action [13]. Mn values were higher in the liver of buffaloes raised on Marajó, both in the dry period (15.86 mg/kg) and in the rainy period (14.86 mg/kg) of the year. An explanation for this fact must be this is due to the higher content of this mineral in the forage of animals raised on the Marajó, mainly in the dry period (401.92 mg/kg), thus causing the accumulation of this mineral in the liver. Even so, these values meet the daily consumption of Mn by adult humans (2.3 mg) [10].

The highest Ba value was observed in the liver of buffaloes reared in the rainy season in cultivated pasture (6.32 mg/kg), and the lowest value (1.42 mg/kg) was detected in the liver of buffaloes reared in the intensive ecosystem. This can be explained by the high levels of Ba in the forage (2.76 mg/kg) and brewery waste (18.29 mg/kg) consumed by the buffaloes during the time studied in both periods of the year.

Cr and Ni showed values lower than 2.0 mg/kg in dry matter (DM) in the liver tissue of buffaloes from the different Amazonian ecosystems studied. As and Li presented values lower than 1.5 mk/kg. The same applied to Pb, Co, Sn and V. This is due to the fact that because in the Brazilian territory, the pastures are seasonal and, as a consequence therefore, there are low forage offers in the dry period of the year, as well as reduced amounts of nutrients. On the other hand, the high volume of water in the rainy season can affect the mineral content in the soil, mainly sandy soils, causing leaching of nutrients due to heavy rains, which consequently make the forage in the pastures have a low content of minerals available for incorporation [58,59,60]. 

Research work with 104 buffaloes located on the Marajó, state of Pará, it was observed that 48.08% of these animals had average hepatic levels of cobalt of 0.36 mg/kg ± 0.33 mg/kg, considered normal in regarding the values for cattle; however, 51.92% of the animals presented values below 0.003 mg/kg, which were considered deficient [45]. In the same research work, mean levels of zinc of 27.05 mg/kg ± 13.12 were verified in the liver of buffaloes, which demonstrates that they had zinc deficiency in the liver [45].

## 5. Conclusions

The different ecosystems of native and cultivated pastures, in the dry and rainy period of the year and one in an intensive ecosystem in the Eastern Amazon, influenced the levels of minerals present in the liver tissue of buffaloes. In general, the liver of buffaloes raised in the ecosystem of Marajó has a higher mineral content and is therefore more nutritious. Even so, the levels of minerals found in the liver of buffaloes raised in other ecosystems meet the daily consumption recommendations for human nutrition.

## Figures and Tables

**Table 1:** Proximate and chemical composition of the diets of Murrah × Mediterranean buffaloes reared intensively and extensively in three types of ecosystems in the Amazon (Brazil) during the dry (DR) and rainy (RS) periods of the year (*n* = 3).

	Extensive							Intensive
	Lower Amazon ^1^		Marajó ^2^		Cultivated Pasture ^3^			Confinement ^4^
	DR	RS	DR	RS	DR		RS	
Proximal Composition (% ms)					Forage	Wet Brewery Waste *		
Dry matter	23.95	24.10	23.87	18.31	23.32	26.12	24.71	39.00
Organic matter	91.53	90.98	89.45	84.86	91.79	96.88	95.35	94.77
Crude protein	7.86	8.72	7.56	8.86	7.73	28.03	9.38	8.27
NDFap	73.23	79.07	68.72	70.91	75.73	55.69	68.96	54.36
ADFap	44.87	54.90	40.05	43.90	55.80	22.55	44.51	38.08
NFC	9.07	1.79	11.79	3.09	6.27	5.16	13.85	29.58
Ether extract	1.36	1.40	1.38	1.99	1.64	8.00	2.06	2.56
TDN	51.59	42.20	56.09	52.5	41.35	72.48	51.93	57.94
Ash	8.47	9.02	10.55	15.14	8.21	3.12	6.17	5.23
Macrominerals (mg/kg)								
Na	111.74	619.37	1288.62	2956.53	941.61	204.80	1885.58	13,166.52
K	18,355.04	11,284.57	16,229.75	14,277.76	18,759.52	3009.34	11,054.25	3009.34
Ca	2971.56	2371.85	2672.26	2624.79	5413.91	4392.73	6536.74	31,684.45
Mg	2034.36	3998.71	2226.14	2111.98	3573.64	2447.88	4839.89	3994.34
P	766.73	790.17	2030.83	806.84	2426.34	8250.37	2772.62	4701.74
S	963.63	816.49	2533.34	2165.16	1253.20	2953.27	1538.24	5565.03
Microminerals (mg/kg)								
Cu	3.19	3.08	**	3.63	6.70	12.85	6.09	53.38
Zn	18.03	29.32	**	37.07	33.98	86.77	31.58	247.11
Fe	106.87	144.36	**	1750.79	203.80	232.18	90.98	1509.62
Mn	100.48	143.52	**	401.92	121.14	46.77	326.46	740.68
Other								
Ba	1.81	4.87	**	10.49	2.76	18.29	3.29	43.49

ote: periods dry (DR); periods rainy (RS). % ms = % dry matter. Na = Sodium, K = Potassium, Ca = Calcium, Mg = Magnesium, P = Phosphorus, S = Sulfur, Cu = Copper, Zn = Zinc, Fe = Iron, Mn = Manganese, Ba = Barium. NDFap = Neutral detergent fiber corrected for ash and protein. ADFap = Acid detergent fiber corrected for ash and protein. NFC = Non-fiber carbohydrates; TDN = Total digestible nutrients. Diets = ^1^ Ecosystem—Lower Amazon—In this breeding ecosystem, buffaloes are raised in the traditional way on pasture, in native pastures in areas subject to flooding. During the collection of pastures, there was also an availability of cultivated grasses Panicum maximum cv. Mombasa and *Bhachiaria brizantha*. ^2^ Ecosystem— Marajó. In this ecosystem, buffaloes are reared in a traditional system, fed exclusively on pasture, with grasses native to areas subject to flooding, such as *Panicum elephantipes, Leersia hexandra and Hymenachne amplexicaulis*. ^3^ Ecosystem—Cultivated Pasture—In this ecosystem, the animals feed on cultivated terra firme pastures (*Panicum maximum* cv. Mombaça and *Bhachiaria humidicola*), in areas originally forested (* the animals received wet brewery waste during the dry period). ^4^ Ecosystem—Confinement—Feed consisted of sorghum silage, soybean meal, wet sorghum premix and commercial feed (high performance core). ** Indicates that there was no data on this parameter.

**Table 2:** Mineral composition in the liver of Murrah × Mediterranean crossbred buffaloes raised intensively and extensively in three types of ecosystems in the Amazon (Brazil), in the dry (DR) and rainy (RS) periods of the year (*n* = 12), and one in lockdown.

Extensive							Intensive					
							Confinement ^4^	EP		*p* Values		
	Lower Amazon ^1^		Marajó ^2^		Cultivated Pasture ^3^				Extensive vs. Intensive	Location (L)	Period (P)	L * P
	DR	RS	DR	RS	DR	RS						
Macrominerals (mg/kg)												
Na	2463.47 ^cd^	2780.81 ^bcd^	3111.65 ^ab^	3497.28 ^a^	2262.74 ^d^	2699.41 ^bcd^	2870.42 ^bc^	131.07	0.6347	<0.0001 (M > S = N)	0.0011 (DR < RS)	0.9099
K	10,454.0 ^c^	11,744.0 ^ab^	10,965.0 ^bc^	11,013.0 ^abc^	12,051.0 ^a^	12,004.0 ^ab^	11,995.0 ^ab^	244.06	0.0212	0.0003	0.0477	0.0211
Ca	672.50 ^a^	676.62 ^a^	717.17 ^a^	747.44 ^a^	476.13 ^b^	477.98 ^b^	677.63 ^a^	28.933	0.1180	<0.0001 (S = M > N)	0.6249	0.8684
Mg	678.42 ^abc^	731.72 ^a^	692.79 ^ab^	697.29 ^ab^	635.01 ^bc^	617.52 ^c^	689.32 ^ab^	15.713	0.419	<0.0001 (S = M > N)	0.3251	0.1039
P	11,714 ^ab^	12,010 ^ab^	11,974 ^ab^	11,469 ^b^	12,140 ^a^	11,695 ^ab^	11,787.00 ^ab^	150.04	0.7726	0.4195	0.0864	0.0180
S	6085.90 ^b^	6703.85 ^a^	6687.72 ^a^	6798.98 ^a^	6059.22 ^b^	6068.00 ^b^	6555.97 ^a^	101.55	0.1628	<0.0001	0.0078	0.0160
Microminerals (mg/kg)												
Cu	18,816.0 ^c^	69,505.8 ^bc^	6235.0 ^c^	6168.3 ^c^	257.96 ^a^	93,784 ^b^	126.14 ^b^	14.873	0.0025	<0.0001	0.0049	<0.0001
Zn	94.06 ^b^	100.38 ^ab^	100.93 ^ab^	116.44 ^a^	102.07 ^ab^	96.36 ^ab^	101.55 ^ab^	4.787	0.9763	0.0652	0.2132	0.1343
Fe	507.62 ^c^	435.94 ^c^	1266.30 ^ab^	1381.29 ^a^	453.97 ^c^	704.39 ^bc^	476.45 ^c^	126.59	0.0249	<0.0001 (M > S = N)	0.3984	0.5330
Mn	13.31 ^ab^	12.50 ^ab^	15.86 ^a^	14.86 ^a^	12.30 ^ab^	12.42 ^ab^	9.71 ^b^	1.05	0.0012	0.0278 (M > N = S)	0.5588	0.8815
Other (mg/kg)												
Ba	4.90 ^abc^	2.04 ^cd^	3.24 ^abcd^	2.62 ^b^	5.62 ^ab^	6.32 ^a^	1.42 ^d^	0.7715	0.0019	0.0018 (N > S = M)	0.1889	0.1227

Note: periods dry (DR); periods rainy (RS). EP = standard error. Na = Sodium, K = Potassium, Ca = Calcium, Mg = Magnesium, P = Phosphorus, S = Sulfur, Cu = Copper, Zn = Zinc, Fe = Iron, Mn = Manganese, Ba = Barium. Different letters on the lines indicate statistical difference (*p* < 0.05). Diets = Diets = ^1^ Ecosystem—Lower Amazon—In this breeding ecosystem, buffaloes are raised in the traditional way on pasture, in native pastures in areas subject to flooding. During the collection of pastures, there was also an availability of cultivated grasses *Panicum maximum* cv. Mombasa and *Bhachiaria brizantha*. ^2^ Ecosystem—Marajó. In this ecosystem, buffaloes are reared in a traditional system, fed exclusively on pasture, with grasses native to areas subject to flooding, such as *Panicum elephantipes, Leersia hexandra and Hymenachne amplexicaulis*. ^3^ Ecosystem—Cultivated Pasture—In this ecosystem, the animals feed on cultivated pastures (*Panicum maximum* cv. Mombaça and *Bhachiaria humidicola*), in areas originally forested (* the animals received wet brewery waste during the dry period). ^4^ Ecosystem—Confinement—Feed consisted of sorghum silage, soybean meal, wet sorghum premix and commercial feed (high performance core).

## Data Availability

The data presented in this study are available upon reasonable request from the corresponding author.

## References

[1] Alarcón-Rojo A., Mota-Rojas D., García-Galicia I., Ramírez-Bribiesca E., Olmos-Hernández A., Guerrero-Legarreta I. (2021). Dark cutting in large ruminants: Effect of management and environmental factors. AGRO Product..

[2] Barboza J.G. (2011). Bondades ecológicas del búfalo de agua: Camino hacia la certificación. Tecnol. Marcha.

[3] Mota-Rojas D., Guerrero-Legarreta I., Pérez-Álvarez J.A., Rosmini M., Napolitano F., Ghezzi M., Fernández-López J., Braghieri A., Viuda M., Bragaglio A., Guerrero-Legarreta I., Napolitano F., Mota-Rojas D., Orihuela A. (2019). Chapter 1: La carne de búfalo de agua en las Américas: Retos y oportunidades. El Búfalo de Agua en las Américas.

[4] Mota-Rojas D., De Rosa G., Mora-Medina P., Braghieri A., Guerrero-Legarreta I., Napolitano F. (2019). Dairy Buffalo Behaviour and Welfare from Calving to Milking. CAB Rev. Perspect. Agric. Vet. Sci. Nutr. Nat. Resour..

[5] Caraballoso A., Borroto Á., Pérez R. (2011). Conducta de búfalos en pastoreo en humedales de Ciego de Ávila, Cuba Behavior of grazing buffaloes in wetlands of Ciego de Ávila, Cuba. Pastos Forrajes.

[6] Barrios-García H., Alva-Pérez J., Corona-Gonzalez B., Obregón-Alvarez D., Martínez-Burnes J., Napolitano F., Mota-Rojas D., Guerrero-Legarreta I., Orihuela A. (2020). Hallazgos recientes del proceso salud-enfermedad en búfalo de agua (*Bubalus bubalis*) Enfermedades bacterianas y parasitarias. El Búfalo de Agua en Latinomerica: Hallazgos Recientes.

[7] Bertoni A., Álvarez-Macías A., Morales-Canela A., Orozco-Corrales C., Mota-Rojas D., Guerrero-Legarreta I., Napolitano F., Mota-Rojas D., Orihuela A. (2019). Chapter 7: Los sistemas de producción de búfalos en el tropico húmedo de América Latina:Un acercamiento desde el enfoque agroecológico. El Búfalo de Agua en las Américas.

[8] Mota-Rojas D., Álvarez-Macías A., Bertoni A., Molina S., José N., López G., Mora-Medina P., Guerrero-Legarreta I., Napolitano F., Guerrero-Legarreta I., Napolitano F., Mota-Rojas D., Orihuela A. (2019). Chapter 8: La importancia de los animales en labores rurales: Tracción, transporte y carga. El Búfalo de Agua en las Américas.

[9] Silva J.A.R., Pantoja M.H.A., Silva W.C., Almeida J.C.F., Noronha R.D.P.P., Barbosa A.V.C., Lourenço-Júnior J.B. (2022). Thermoregulatory reactions of female buffaloes raised in the sun and in the shade, in the climatic conditions of the rainy season of the Island of Marajó, Pará, Brazil. Front. Vet. Sci..

[10] Soetan K.O., Olaiya C.O., Oyewole O.E. (2010). The importance of mineral elements for humans, domestic animals and plants: A review. Afr. J. Food Sci..

[11] Kabata-Pendias A., Szteke B. (2012). Trace elements in geo-and biosphere. The Institute of Soil Science and Plant Cultivation, Puławy, Poland.

[12] Kicińska A., Glichowska P., Mamak M. (2019). Micro-and macroelement contents in the liver of farm and wild animals and the health risks involved in liver consumption. Environ. Monit. Assess..

[13] Parinet J., Royer E., Saint-Hilaire M., Chafey C., Noël L., Minvielle B., Dervilly-Pinel G., Engel E., Guérin T. (2018). Classification of trace elements in tissues from organic and conventional French pig production. Meat Sci..

[14] Ribeiro D.M., Mourato M.P., Almeida A.M. (2019). Assessing mineral status in edible tissues of domestic and game animals: A review with a special emphasis in tropical regions. Trop. Anim. Health Prod..

[15] Silva F.L., Oliveira-Júnior E.S., Silva M.H.M.E., López-Alonso M., Pierangeli M.A.P. (2022). Trace Elements in Beef Cattle: A Review of the Scientific Approach from One Health Perspective. Animals.

[16] Underwood E.J. (1981). Los Minerales en la Nutrición del Ganado.

[17] Tokarnia C.H., Peixoto P.V., Barbosa J.D., Brito M.F., Dobereiner J. (2010). Deficiências Minerais em Animais de Produção.

[18] Institute of Medicine (2001). IOM Reference Dietary Intake for Vitamin A, Vitamin K, Arsenic, Boron, Chromium, Copper, Iodine, Iron, Manganese, Molybdenum, Nickel, Silicon, Vanadium and Zinc.

[19] Silva J.A.R., Garcia A.R., Almeida A.M., Bezerra A.S., Lourenço-Júnior J.B. (2021). Water buffalo production in the Brazilian Amazon Basin: A review. Trop. Anim. Health Prod..

[20] Silva J.A., Rodrigues L.S., Lourenço-Júnior J.D., Alfaia C.M., Costa M.M., Castro V.C., Bezerra A.S., Almeida A.M., Prates J.A. (2022). Total Lipids, Fatty Acid Composition, Total Cholesterol and Lipid-Soluble Antioxidant Vitamins in the *Longissimus lumborum* Muscle of Water Buffalo (*Bubalus bubalis*) from Different Production Systems of the Brazilian Eastern Amazon. Animals.

[21] Júnior H.B.M., Oliveira Morais F.I., Neto J.F.T., Couto W.S., Viégas I.D.J.M. (2000). Nutrientes limitantes em pastagens nativas de Panicum zizanoides HBK e Axonopus affinis em um plintossolo da ilha de Marajó, Pará. Rev. Ciencias Agr. Amaz. J. Agric. Env. Sci..

[22] Lourenço Júnior J.B., Garcia A.R. (2006). Produção animal no Bioma Amazônico: Atualidades e perspectivas. Rev. Bras. Zootec..

[23] Köppen W., Geiger R. (1928). Klimate der Erde. Justus Perthes, Gotha. file:///C:/Users/MDPI/Downloads/10.1515_9783111491530-fm.pdf.

[24] INMET: Instituto Nacional de Meteorológicos Dados Meteorológicos. https://mapas.inmet.gov.br/.

[25] Detmann E., Souza M.A., Valadares Filho S.C., Queiroz A.C., Berchielli T.T., Saliba E.O.S. (2012). Métodos Para Análise de Alimentos–INCT–Ciência Animal.

[26] Sniffen C.J., O’Connor J.D., Van Soest P.J. (1992). A net carbohydrate and protein system for evaluating cattle diets: II. carbohydrate and protein availability. J. Anim. Sci..

[27] Roselli C., Desideri D., Meli M.A., Fagiolino I., Feduzi L. (2016). Essential and toxic elements in meat of wild birds. J. Toxicol. Environ. Health.

[28] Statistical Analysis System (SAS) (2020). SAS/STAT 9. User’s Guide.

[29] Langaro A., Tomkiel L.C.E., Mazon L.R., Carniel T.K., Dalcanton F., Zanetti M. (2020). Determination of the quantity of sodium and potassium in organic and industrialized baby papers. Res. Soc. Dev..

[30] Ribeiro D.M., Scanlon T., Kilminster T., Martins C., Greeff J., Milton J., Oldham C., Freire J.P.B., Mourato M.P., De Almeida A.M. (2020). Mineral profiling of muscle and hepatic tissues of Australian Merino, Damara and Dorper lambs: Effect of weight loss. J. Anim. Physiol. Anim. Nutr..

[31] Domingues B.C., Ribeiro T.R., Neves A.L., Nascimento-Júniora N.M. (2023). Suplementos Alimentares: Aspectos Químicos e Aplicações de Macro e Micronutrientes. Rev. Virtual Quim..

[32] Suttle N.F. (2010). Mineral Nutrition of Livestock.

[33] Camarao A.P., Lourenco-Júnior J.B., Dutra S., Hornick J.L., Silva M.B. (2004). Grazing buffalo on flooded pastures in the Brazilian Amazon region: A review. Trop. Grassl..

[34] Grüdtner V.S., Weingrill P., Fernandes A.L. (1997). Aspectos da absorção no metabolismo do cálcio e vitamina D. Rev. Bras. Reumatol..

[35] Tajik H., Rezaei S.A., Alamouti M.R.P., Moradi M., DalirNaghadeh B. (2010). Mineral contents of muscle (Longissimus dorsi thoracis) and liver in river buffalo (*Bubalus bubalis*). J. Muscle Foods.

[36] Niedziolka R., Pieniak-Lendzion K., Horoszewicz E. (2010). Content of chemical elements in muscular tissue and liver of male kids and ram lambs in central-eastern Poland. J. Elem..

[37] Alturiqi A.S., Albedair L.A. (2012). Evaluation of some heavy metals in certain fish, meat and meat products in Saudi Arabian markets. Egypt. J. Aquat. Res..

[38] Hernández-Castellano L.E., Hernandez L.L., Sauerwein H., Bruckmaier R.M. (2017). Endocrine and metabolic changes in transition dairy cows are affected by prepartum infusions of a serotonin precursor. J. Dairy Sci..

[39] Khristoforova N.K., Tsygankov V.Y., Lukyanova O.N., Boyarova M.D. (2016). The Kuril Islands as a potential region for aquaculture: Trace elements in chum salmon. Environ. Pollut..

[40] Lin K.C., Cross H.R., Johnson H.K., Breidenstein B.C., Randecker V., Field R.A. (1988). Mineral composition of lamb carcasses from the United States and New Zealand. Meat Sci..

[41] Özcelik R., Bruckmaier R.M., Hernández-Castellano L.E. (2017). Prepartum daylight exposure increases serum calcium concentrations in dairy cows at the onset of lactation. J. Anim. Sci..

[42] Taniguchi C.N., Dobbs J., Dunn M.A. (2017). Heme iron, non-heme iron, and mineral content of blood clams (*Anadara* spp.) compared to Manila clams (*V. philippinarum*), Pacific oysters (*C. gigas*), and beef liver (*B. taurus*). J. Food Compos. Anal..

[43] Lobo A.S., Tramonte V.L.C. (2004). Efeitos da suplementação e da fortificação de alimentos sobre a biodisponibilidade de minerais. Rev. De Nutr..

[44] Padovani R.M., Amaya-Farfán J., Colugnati F.A., Domene S.M. (2006). Dietary reference intakes: Application of tables in nutritional studies. Rev. De Nutr.-Camp..

[45] Pinheiro C.P., Bomjardim H.A., Andrade S.J., Faial K.C., Oliveira C.M., Barbosa J.D. (2011). Níveis de fósforo, cobre, cobalto e zinco em bubalinos (*Bubalus bubalis*) na Ilha de Marajó, Estado do Pará. Pesqui. Veterinária Bras..

[46] Maynard L.A., Looslie J.K., Hintz H.F., Warner R.G. (1984). Nutrição Animal.

[47] Conrad J.H., Mcdowell L.R., Ellis G.L., Loosli J.K. (1985). Minerais Para Ruminantes em Pastejo em Regiões Tropicais, Campo Grande, M.S., EMBRAPA-CNPGC, p. 90. https://pdf.usaid.gov/pdf_docs/pnabc098.pdf.

[48] Tokarnia C.H., Döbereiner J., Peixoto P.V. (2000). Deficiências minerais em animais de fazenda, principalmente bovinos em regime de campo. Pesqui. Veterinária Bras..

[49] Wintergerst E.S., Maggini S., Hornig D.H. (2007). Contribution of selected vitamins and trace elements to immune function. Ann. Nutr. Metab..

[50] Dorton K.L., Engle T.E., Hamar D.W., Siciliano P.D., Yemm R.S. (2003). Effects of copper source and concentration on copper status and immune function in growing and finishing steers. Anim. Feed. Sci. Technol..

[51] Fox P.L. (2003). The copper-iron chronicles: The story of an intimate relationship. Biometals.

[52] Spears J.W. (2000). Micronutrients and immune function in cattle. Proc. Nutr. Soc..

[53] Pereira W.L.A., Cardoso E.C. (2009). Aspectos histológicos da osteoporose em bubalinos e a condição físico-química óssea e do cobre hepático. Rev. Ciências Agrárias.

[54] Bonaventura P., Benedetti G., Albarède F., Miossec P. (2015). Zinc and its role in immunity and inflammation. Autoimmun. Rev..

[55] Calder P.C., Carr A.C., Gombart A.F., Eggersdorfer M. (2020). Reply to “Comment on: Optimal Nutritional Status for a Well-Functioning Immune System Is an Important Factor to Protect against Viral Infections. Nutrients.

[56] Yip R. (2000). Significance of an abnormally low or high hemoglobin concentration during pregnancy: Special consideration of iron nutrition. Am. J. Clin. Nutr..

[57] Beard J. (2001). Iron-Deficiency Anemia: Reexamining the Nature and Magnitude of the Public Health Problem: Iron Biology in Immune Function, Muscle Metabolism and Neuronal Functioning. J. Nutr..

[58] Vormann J. (2003). Magnesium: Nutrition and metabolism. Mol. Asp. Med..

[59] Khan Z.I., Ashraf M., Valeem E.E., Ahmad K., Danish M. (2007). Pasture concentration of minerals in relation to the nutrient requirementes of farm livestock Pakistan. J. Bot..

[60] Silvanus S.K., Veronica N., Hudson N., Isaac J., Fredrick O. (2014). Avaliação das deficiências minerais entre áreas de pastagem no Condado de Uasin Gishu, Quênia. Int. J. Food Sci. Nutr..

